# The importance of advising in an accelerated pathway program

**DOI:** 10.1080/10872981.2024.2430053

**Published:** 2024-11-28

**Authors:** Joan Cangiarella, Elisabeth Cohen

**Affiliations:** Grossman School of Medicine, New York University, New York, NY, USA

**Keywords:** Advisors, mentoring, accelerated MD program, 3 year MD pathway, residency selection, undergraduate medical education

## Abstract

In the last decade, there has been tremendous growth in the number of accelerated three-year medical pathway programs. The needs of accelerated pathway students are different from traditional students, and a robust mentoring program should be developed to address specific issues and guarantee student success. We describe a unique approach to the development of a mentoring program for accelerated three-year MD students at New York University Grossman School of Medicine.

## Introduction

In the last decade, there has been tremendous growth in the number of accelerated medical pathway programs. The Consortium of Accelerated Medical Pathway Programs [1] started in 2014 with funding by the Josiah Macy Foundation has grown to now include over 30 medical school members. The accelerated three-year MD (3YMD) pathway at NYU Grossman School of Medicine (NYUGSOM) began in 2013 when a curricular redesign shortened the pre-clerkship time to 18 months. This enabled the formation of an accelerated three-year MD pathway for students who had decided early on their career path to apply to any of 21 residencies at NYU Langone Health (NYULH) and, if accepted and completed all requirements, to graduate in three years. With an accelerated pace, concerns related to student wellness due to the pace of the curriculum, professional identity development, and certainty with career choice are more prevalent for 3YMD students, and the need to take licensing exams with less study time than traditional students, and the achievement of competency in a shortened timeframe required the development of a robust advising program. The advising program was critical to the success of the accelerated 3YMD pathway.

## Program structure

At NYUGSOM, students are first accepted into the medical school. Once accepted they have the opportunity to apply into the accelerated 3YMD program at matriculation, in the spring of the first or second year of medical school, or after completion of the PHD portion of the Medical Science Training Program (MSTP). Interested students in good standing complete an application describing their career choice and are interviewed by the residency program. Since the accelerated 3YMD pathway at NYUGSOM also has a directed pathway to any of the 21 residency programs at NYU Langone Health, it is the residency program that makes the final decision regarding acceptance to the accelerated 3YMD pathway. The curriculum for the three- and four-year students is identical during the pre-clerkship and clerkship blocks. The 3YMD students complete 130 weeks of medical school (only 19 weeks less than the traditional four-year program), including research, sub-internship, critical care, and elective rotations, and are eligible for NY state medical licenses. 3YMD students have the option to apply out for residency through the National Resident Matching Program, apply to switch specialties at NYULH within the pathway, or shift to the 4- year pathway. Approximately 15% of the class at NYUGSOM are accelerated 3YMD students. From 2016 to 2024, 195 students have entered the program. Nineteen students have transitioned to the four-year program, 6 of them due to professional concerns, and 13 due to their own decisions (change in specialty choice or a personal reason). One hundred and seventy-six students have graduated and of these 11 have switched specialties but remained in the 3YMD program. Studies have shown that our 3YMD students perform similarly to our 4YMD students in medical school and early residency [2,3].

## Program implementation

Successful implementation of an accelerated program requires unwavering commitment from the Dean and other leaders of the medical school [4,5]. A larger rather than smaller program has the advantage of facilitating bonding and peer support among accelerated students. Given the novelty and accelerated pace, it was realized early on that intensive mentoring was needed for success.

## Advising program

There are several factors to consider in the development of a advising program for accelerated 3YMD students. They need to have early clinical experiences that confirm their career choice or introduce them to other specialties that better align with their long-term aspirations. They must have a high degree of professionalism and are assessed through evaluations and reported complaints. Students are expected to maintain academic honesty and integrity and contribute to a positive learning environment with respectful and inclusive behaviors.

A multi-pronged approach provides extra advising to accelerated 3YMD students. Advisors provide specific, practical advice to students in helping them navigate through the accelerated program. In creating this advising program, we needed it to serve the needs of accelerated 3YMD students but at the same time not duplicate or destroy what we already had in place. In addition to career and peer advisors that are assigned to all students (three-year and four-year students), they have a departmental advisor upon acceptance into the residency program, rather than later in medical school (as seen in the four-year program), and a dedicated 3YMD pathway advisor. The career advisor and the 3YMD pathway advisor receive stipends.

The career advisors review academic performance and professional growth, assist in the residency application process, and have social activities to integrate students in all classes. The peer advisor is a student or resident who is either senior to or a graduate of the 3YMD pathway usually in the same medical specialty. At NYUGSOM, the peer mentorship program is unique in that it provides student advisors across all specialties and thus has the availability of a large group of peer advisors. This differs from other accelerated 3YMD programs which may only focus on primary care specialties and have a much smaller cohort of students to serve as peer advisors.

The departmental advisor integrates 3YMD students into the department, introduces them broadly to clinical and research opportunities and potential mentors, and facilitates attendance at conferences and social events. The goal is to help solidify their career choice and decision to stay for residency, or to apply to outside residencies if they prefer. If the student stays at NYUGSOM for residency training, some departmental advisors will continue to mentor the student through their residency, facilitating a continuum of undergraduate and post-graduate medical education.

It is the accelerated 3YMD pathway advisor role that is unique and critical for any medical school considering the development of an accelerated pathway. This position required a faculty member who was already experienced in advising who could commit to a .2FTE position. The skills necessary for this advisor includes being knowledgeable, being a good communicator, an active listener, a problem-solver, and an action-oriented personality. The accelerated 3YMD advisor is responsible for all students in the pathway and meets three times a year individually with each student and six times a year in group sessions. Students in an innovative program benefit from additional support and guidance. The accelerated 3YMD pathway advisor understands the curricular requirements for graduation and the options available. The advisor can ensure that questions related to the program in general and concerns of individual students are addressed promptly. Optimal communication is facilitated by frequent regularly scheduled meetings in person, one on one, on acceptance and then quarterly, as well as monthly for all 3YMD students. The advisor reviews schedules, timing of board preparation and examinations, selection of electives, and completion of requirements for graduation. The accelerated 3YMD advisor encourages early broad shadowing both in and out of their chosen specialty, as some students may have relatively narrow experiences in their field and may find other specialties better aligned with their long-term goals. The accelerated 3YMD advisor helps students who decide to change specialties, apply for an outside residency or become 4-year students. This advisor also tries to support students with professional and personal issues, and arrange for additional resources as needed. While the accelerated 3YMD advisor does not have access to grades, they participate in bimonthly meetings where academic concerns related to students are relayed. The 1:1 meetings with students enables a relationship that encourages students to be transparent and open regarding their performance and the need for help. The accelerated 3YMD advisor serves as a liaison between the student, the Director of the pathway, and the Deans for Medical Education and Student Affairs at scheduled meetings and as needed. By serving in this liaison role, with no oversight of grades students are more willing to discuss issues and the accelerated 3YMD advisor can recommend resources or ask the 3YMD director or Deans to intervene in order to help students to adjust their learning strategies in order to succeed or provide guidance when students need to remediate.

One of the best practices that has been translated across to the four-year pathway at NYUGSOM has been the role of the departmental advisor. As noted above, a departmental advisor is assigned at the time of admission to the accelerated 3YMD pathway to explore departmental opportunities for students and to provide experiences to help students be certain of their career choice. While this advisor was traditionally assigned to students in other pathways closer to their residency application process, the success of this change in the advising program for 3YMD accelerated student has led to the assignment earlier at the time of expressed interest in a residency specialty. This allows all students to solidify their specialty choice sooner and focus on personalizing their electives towards their specialty choice.

**Figure 1.**
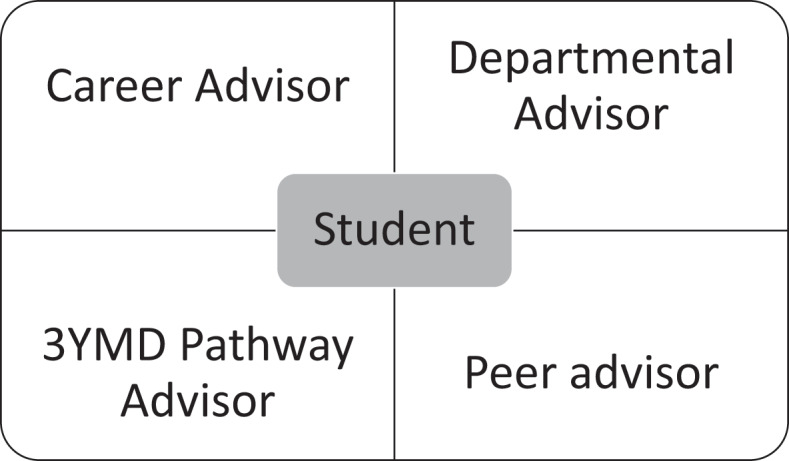
A student-centered approach to advising in an accelerated 3YMD pathway.

Legend: Multiple advisors in an accelerated 3YMD Pathway help to guarantee student success.

Key in this multi-faceted approach to advising is having a student-centered approach (Figure 1). The student takes the lead in working with multiple advisors. The career advisor helps instruct students on the role of the different advisors. During orientation, a presentation to students outlines the roles of the different advisors. In addition to this group orientation session, the career advisor meets individually with new students in the first few months of medical school to give each student individualized guidance on approaching the different advisors. Having several advisors with different roles in an accelerated 3YMD pathway assures that students are successful in medical school.
